# Elevated cerebral pulsatility is associated with cerebrospinal fluid biomarkers of neurodegeneration in cognitively unimpaired adults

**DOI:** 10.1002/alz70856_104246

**Published:** 2025-12-24

**Authors:** M. Erin Moir, Brandon G. Fico, Sarean Harmoni A. Gaynor‐Metzinger, Nicole A. Loggie, Alexander M. Norby, Anna J. Howery, Gwendlyn Kollmorgen, Henrik Zetterberg, Kaj Blennow, Oliver Wieben, Sterling C Johnson, Jill N. Barnes

**Affiliations:** ^1^ University of Wisconsin‐Madison, Madison, WI, USA; ^2^ Florida Atlantic University, Boca Raton, FL, USA; ^3^ Roche Diagnostics GmbH, Penzberg, Germany; ^4^ Hong Kong Center for Neurodegenerative Diseases, Hong Kong, Science Park, China; ^5^ University of Gothenburg, Mölndal, Sweden; ^6^ Sahlgrenska University Hospital, Mölndal, Sweden; ^7^ UCL Institute of Neurology, London, United Kingdom; ^8^ UK Dementia Research Institute at UCL, London, United Kingdom; ^9^ University of Wisconsin School of Medicine and Public Health, Madison, WI, USA; ^10^ Alzheimer's Disease Research Center, University of Wisconsin‐Madison, Madison, WI, USA

## Abstract

**Background:**

Cerebrovascular dysregulation, including elevated cerebral pulsatility, contributes to the increased risk of Alzheimer's disease (AD) with advancing age. However, the mechanisms by which elevated cerebral pulsatility contributes to declining brain health in humans remain unclear. The transmission of pulsatile blood flow into the brain's microcirculation may damage the blood brain barrier resulting in dysregulation of amyloid‐β (Aβ) and tau levels, neuronal injury, and synaptic dysfunction. Therefore, this study tested the hypothesis that elevated cerebral pulsatility is associated with cerebrospinal fluid (CSF) markers of neurodegeneration in cognitively unimpaired adults.

**Methods:**

CSF samples (lumbar puncture) and 3T magnetic resonance imaging (MRI) were completed in 207 cognitively unimpaired middle‐aged and older adults (144 females, 63±8 years of age). CSF samples were analyzed for markers of neurodegeneration including α‐synuclein (pre‐synaptic), Aβ_1‐42_ (Aβ42), neurofilament light (NfL), neurogranin, phosphorylated tau (pTau), and total tau (tTau) using the NeuroToolKit, a panel of exploratory robust prototype assays (Roche Diagnostics International Ltd). Intracranial blood flow was quantified using 4D flow MRI in the internal carotid arteries (ICA), middle cerebral arteries (MCA), vertebral arteries (VA), and basilar artery (BA), and cerebral pulsatility index (PI) was calculated as maximum flow – minimum flow/mean flow.

**Results:**

In cognitively unimpaired adults, right ICA PI was positively associated with NfL (β=127±17 pg/mL, R^2^=0.22, *p* <0.01) and tTau (β=139±29 pg/mL, R^2^=0.10, *p* <0.01). Right MCA PI was positively associated with α‐synuclein (β=68±19 pg/mL, R^2^=0.06, *p* <0.01) and tTau (β=87±21 pg/mL, R^2^=0.08, *p* <0.01). Further, PI was positively associated with NfL in the left ICA (β=123±16 pg/mL, R^2^=0.23, *p* <0.01), left VA (β=44±9 pg/mL, R^2^=0.10, *p* <0.01), and basilar artery (β=52±12 pg/mL, R^2^=0.08, *p* <0.01). These associations persisted when age was included (all *P*≤0.047). Biological sex influenced the association between left VA PI and NfL whereby males demonstrated stronger associations than females (*p* = 0.01). All other associations did not differ between males and females (all *P*>0.09).

**Conclusion:**

Overall, elevated cerebral PI, measured with 4D flow MRI, was associated with CSF biomarkers of neurodegeneration and synaptic dysfunction in cognitively unimpaired middle‐aged and older adults. As such, cerebral pulsatility may represent an early biomarker for AD risk and interventions that target cerebral pulsatility may mitigate neurodegeneration.

